# Smoothness Parameter of Power of Euclidean Norm

**DOI:** 10.1007/s10957-020-01653-6

**Published:** 2020-03-27

**Authors:** Anton Rodomanov, Yurii Nesterov

**Affiliations:** 1 ICTEAM, Catholic University of Louvain, Louvain-la-Neuve, Belgium; 2 Center for Operations Research and Economics, Catholic University of Louvain, Louvain-la-Neuve, Belgium

**Keywords:** Hölder continuity, Polynomials, Optimal constants, 26A16, 46G05, 11C08

## Abstract

In this paper, we study derivatives of powers of Euclidean norm. We prove their Hölder continuity and establish explicit expressions for the corresponding constants. We show that these constants are optimal for odd derivatives and at most two times suboptimal for the even ones. In the particular case of integer powers, when the Hölder continuity transforms into the Lipschitz continuity, we improve this result and obtain the optimal constants.

## Introduction

Starting from the paper [1], there has been an increasing interest in the *cubic regularization* of Newton’s method (see, for example, [2–8]), which has some attractive global worst-case complexity guarantees. The main idea of this method is to approximate the objective function with its second-order Taylor approximation, add to it the cube of Euclidean norm with certain coefficient and then minimize the result to obtain a new point.

A natural generalization of this approach consists in considering a general high-order Taylor approximation together with a certain high-order power of Euclidean norm as a regularizer. This leads to *tensor methods* [9–12] that have recently gained their popularity after it was shown in [13] that one step of the third-order tensor method for minimizing convex functions is comparable with that of the cubic Newton method.

For some applications, involving functions with Hölder continuous derivatives, it may also be reasonable to regularize the models with *fractional* degrees of the Euclidean norm, as discussed in [14, 15].

The efficiency of all the aforementioned methods strongly depends on our possibilities in solving the corresponding auxiliary problems that arise at each iteration. Therefore, it is important to be able to quickly solve minimization problems regularized by powers of Euclidean norm.

Two of the most important characteristics of the objective function that influence the convergence rate of minimization algorithms are the constants of uniform convexity and Hölder continuity of derivatives. It is thus important to know these parameters for powers of Euclidean norm in order to justify the convergence rates of the related minimization algorithms.

The uniform convexity of powers of Euclidean norm was first investigated in [16], where the authors obtained optimal constants for all *integer* powers. This result was then generalized to arbitrary *real* powers in [17, Lemma 5]. Thus, the question of uniform convexity is completely solved.

The question of the Hölder continuity of derivatives of powers of Euclidean norm is more subtle. There exist only partial results for some special powers. For example, for any real power between one and two, the Hölder continuity of the first derivative follows from the duality between uniform convexity and Hölder smoothness (see [18, Lemma 1]). For any real power between two and three, the Hölder continuity of the second derivative has recently been proved in [17, Example 2], where some suboptimal constants have been obtained. However, there are currently no general results for an arbitrary power.

Thus, establishing Hölder continuity of derivatives of powers of Euclidean norm and estimating the corresponding constants is still an open problem and constitutes the main topic of this work.

This paper is organized as follows. In Sect. 2, we introduce notation and recall important facts on the norm of symmetric multilinear operators.

In Sect. 3, we derive a general formula for derivatives of powers of Euclidean norm (Theorem 3.1). The main object in this formula is a certain family of recursively defined polynomials (Definition 3.1). We give the corresponding definition and provide several examples.

In Sects. 4 and 5, we study these polynomials in more detail. We establish useful identities and prove several important properties such as symmetry (Proposition 4.1), nonnegativity (Proposition 4.3) and monotonicity (Proposition 4.4). Section 5 is devoted to estimating the Hölder constants of the polynomials. The main results in this section are Theorems 5.1 and 5.2.

In Sect. 6, we apply the auxiliary results obtained in the previous sections for proving Hölder continuity of derivatives of powers of Euclidean norm. Namely, in Theorem 6.1, we derive a lower bound for the possible values of Hölder constants. In Theorem 6.2, we prove Hölder continuity of the derivatives along the lines passing through the origin. Finally, in Theorem 6.3, we extend this result onto the whole space and discuss the optimality of the constants.

Finally, in Sect. 7, we show how to improve our general result for integer powers, when the Hölder condition corresponds to the Lipschitz condition.

## Notation and Generalities

In this text, $\mathbb {E}$ is a finite-dimensional real vector space. Its dual space, composed of all linear functionals on $\mathbb {E}$, is denoted by $\mathbb {E}^*$. The value of a linear functional $s \in \mathbb {E}^*$, evaluated at a point $x \in \mathbb {E}$, is denoted by $\langle s, x \rangle$. To introduce a Euclidean norm $\Vert \cdot \Vert$ on $\mathbb {E}$, we fix a self-adjoint positive definite operator $B : \mathbb {E}\rightarrow \mathbb {E}^*$ and define $\Vert x \Vert := \langle B x, x \rangle ^{1/2}$.

For a function $f : G \rightarrow \mathbb {R}$, defined on an open set *G* in $\mathbb {E}$, and for an integer $p \ge 0$, the *p*th derivative of *f*, if exists, is denoted by $D^p f$. This derivative is a mapping from *G* to the space of symmetric *p*-multilinear forms on $\mathbb {E}$.

Let *L* be a *p*-multilinear form on $\mathbb {E}$. Its value, evaluated at $h_1, \ldots , h_p \in \mathbb {E}$, is denoted by $L[h_1, \ldots , h_p]$. When $h_1 = \cdots = h_p = h$ for some $h \in \mathbb {E}$, we abbreviate this as $L[h]^p$. The norm of *L* is defined in the standard way:

$$\begin{aligned} \Vert L \Vert := \max \limits _{ \Vert h_1 \Vert = \cdots = \Vert h_p \Vert = 1} | L[h_1, \ldots , h_p] |. \end{aligned}$$

If the form *L* is symmetric, it is known that the maximum in the above definition can be achieved when all the vectors are the same:

1 $$\begin{aligned} \Vert L \Vert = \max \limits _{ \Vert h \Vert = 1} | L[h]^p | \end{aligned}$$

(see, for example, Appendix 1 in [19]).

For $q \in \mathbb {R}$, by $f_q : \mathbb {E}\rightarrow \mathbb {R}$ we denote the *q*th power of the Euclidean norm:

$$\begin{aligned} f_q(x) := \Vert x \Vert ^q. \end{aligned}$$

The main goal of this paper is to establish that, for any integer $p \ge 0$ and any real $\nu \in [0, 1]$, the *p*th derivative of $f_{p+\nu }$ is $\nu$-Hölder continuous:

$$\begin{aligned} \Vert D^p f_{p+\nu }(x_2) - D^p f_{p+\nu }(x_1) \Vert \le A_{p, \nu } \Vert x_2 - x_1 \Vert \end{aligned}$$

for all $x_1, x_2 \in \mathbb {E}$, where $A_{p, \nu }$ is an explicit constant dependent on *p* and $\nu$.

## Derivatives of Powers of Euclidean Norm

We start with deriving a general formula for derivatives of the function $f_q$. The main objects in this formula are univariate polynomials, defined below.

### Definition 3.1

For each integer $p \ge 0$ and each $q \in \mathbb {R}$, we define a polynomial $g_{p, q} : \mathbb {R}\rightarrow \mathbb {R}$ as follows. When $p=0$, we set $g_{p, q}(\tau ) := 1$. For all other $p \ge 1$,

$$\begin{aligned} g_{p, q}(\tau ) := (1-\tau ^2) g_{p-1, q}'(\tau ) + (q-p+1) \tau g_{p-1, q}(\tau ). \end{aligned}$$

Each polynomial $g_{p, q}$ is a combination of the previous polynomial $g_{p-1, q}$ and its derivative $g_{p-1, q}'$. The first five polynomials can be written explicitly:

$$\begin{aligned} g_{0, q}(\tau )= & {} 1, \quad g_{1, q}(\tau ) \; = q \tau , \quad g_{2, q}(\tau ) \; = q [ (q-2) \tau ^2 + 1 ], \\ g_{3, q}(\tau )= & {} q (q-2) [ (q-4) \tau ^3 + 3 \tau ], \\ g_{4, q}(\tau )= & {} q (q-2) [ (q-4) (q-6) \tau ^4 + 6 (q-4) \tau ^2 + 3 ]. \end{aligned}$$

Let us now describe how derivatives of $f_q$ are related to polynomials $g_{p, q}$.

### Theorem 3.1

For any real $q \in \mathbb {R}$, the function $f_q$ is *p* times differentiable for all integer $0 \le p < q$. The corresponding derivatives are

2 $$\begin{aligned} D^p f_q(x)[h]^p = \Vert x \Vert ^{q-p} g_{p, q}(\tau _h(x)), \end{aligned}$$

where $h \in \mathbb {E}$ is an arbitrary unit vector and

3 $$\begin{aligned} \tau _h(x) := {\left\{ \begin{array}{ll} \frac{\langle B x, h \rangle }{\Vert x \Vert }, &{}\quad \mathrm{if}~x \ne 0, \\ 0, &{}\quad \mathrm{if}~x = 0. \end{array}\right. } \end{aligned}$$

### Proof

Note that $f_q$ is infinitely differentiable on $\mathbb {E}{\setminus } \{0\}$ since its restriction on this set is a composition of two infinitely differentiable functions, namely the quadratic function $\mathbb {E}{\setminus } \{0\} \rightarrow \mathbb {R}: x \mapsto \Vert x \Vert ^2 = \langle B x, x \rangle$ and the power function $]0, +\infty [ \rightarrow \mathbb {R}: t \mapsto t^{q/2}$. Hence, we only need to prove that $f_q$ is also *p* times differentiable at the origin for any $0 \le p < q$, and that (2) holds.

We proceed by induction. The case $p=0$ is trivial since, by definition, the zeroth derivative of a function is the function itself, while $g_{0, q}(\tau ) = 1$ for any $\tau \in \mathbb {R}$. Let us assume that $p \ge 1$, and the claim is proved for $p' := p-1$.

First, let us justify (2) for any $x \in \mathbb {E}{\setminus } \{0\}$. By the induction hypothesis,

$$\begin{aligned} D^{p-1} f_q(x)[h]^{p-1} = \Vert x \Vert ^{q-p+1} g_{p-1, q}(\tau _h(x)) \end{aligned}$$

for all $x \in \mathbb {E}$. On differentiating, we obtain that

$$\begin{aligned} D \Vert \cdot \Vert (x)[h] = \tau _h(x), \quad D \tau _h(x)[h] = \frac{1 - \tau _h^2(x)}{ \Vert x \Vert } \end{aligned}$$

for all $x \in \mathbb {E}{\setminus } \{0\}$, and hence,

$$\begin{aligned} D^p f_q(x)[h]^p= & {} \Vert x \Vert ^{q-p+1} g_{p-1, q}'(\tau _h(x)) \frac{1 - \tau _h^2(x)}{ \Vert x \Vert } \\&+\, (q-p+1) \Vert x \Vert ^{q-p} \tau _h(x) g_{p-1, q}(\tau _h(x)) \\= & {} \Vert x \Vert ^{q-p} [ (1 - \tau _h^2(x)) g_{p-1, q}'(\tau _h(x)) + \tau _h(x) g_{p-1, q}(\tau _h(x)) ] \\= & {} \Vert x \Vert ^{q-p} g_{p, q}(\tau _h(x)), \end{aligned}$$

where the last equality follows from Definition 3.1.

Now let us show that $f_q$ is also *p* times differentiable at the origin with $D^p f_q(0) = 0$. [This is what (2) says when $x=0$.] By our inductive assumption, we already know that $D^{p-1} f_q(0) = 0$. Therefore, according to the definition of derivative, it remains to show that $\lim _{x \rightarrow 0; x \ne 0} \frac{ \Vert D^{p-1} f_q(x) \Vert }{\Vert x \Vert } = 0$, or, equivalently, in view of (1), that

$$\begin{aligned} \lim \limits _{x \rightarrow 0; x \ne 0} \max \limits _{\Vert h \Vert = 1} \frac{ | D^{p-1} f_q(x)[h]^{p-1} | }{ \Vert x \Vert } = 0. \end{aligned}$$

Applying our inductive assumption, we obtain that

4 $$\begin{aligned} \max \limits _{\Vert h \Vert = 1} \frac{ | D^{p-1} f_q(x)[h]^{p-1} | }{\Vert x \Vert } = \Vert x \Vert ^{q-p} \max \limits _{\Vert h \Vert = 1} |g_ {p-1, q}(\tau _h(x))| \end{aligned}$$

for all $x \in \mathbb {E}{\setminus } \{0\}$. Since $p < q$, we have $\Vert x \Vert ^{q-p} \rightarrow 0$ as $x \rightarrow 0$. Thus, we need to show that $|g_{p-1, q}(\tau _h(x))|$ is uniformly bounded for all $x \in \mathbb {E}$ and all unit $h \in \mathbb {E}$. Indeed, by Cauchy–Schwartz inequality, we have $|\tau _h(x)| \le 1$. Hence, $|g_{p-1, q}(\tau _h(x))| \le \max _{[-1, 1]} |g_{p-1, q}|$. The right-hand side in the above inequality is finite, since a continuous function always achieves its maximum on a compact interval. $\square$

## Main Properties of Polynomials

Let us study the polynomials $g_{p, q}$ introduced in Definition 3.1. Our first observation is that $g_{p, q}$, as a function, is always either even or odd.

### Proposition 4.1

For any integer $p \ge 0$, and any $q \in \mathbb {R}$, $g_{p, q}$ has the same parity as *p*, i.e., $g_{p, q}(-\tau ) = (-1)^p g_{p, q}(\tau )$ for all $\tau \in \mathbb {R}$.

### Proof

Easily follows from Definition 3.1 by induction.$\square$

Next we establish identities with the first and second derivatives of $g_{p, q}$.

### Lemma 4.1

For any integer $p \ge 1$, and any $q, \tau \in \mathbb {R}$,

5 $$\begin{aligned} g_{p, q}'(\tau ) = (1-\tau ^2) g_{p-1, q}''(\tau ) + (q-p-1) \tau g_{p-1, q}'(\tau ) + (q-p+1) g_{p-1, q}(\tau ). \end{aligned}$$

### Proof

Follows from Definition 3.1 using standard rules of differentiation.$\square$

### Lemma 4.2

For any integer $p \ge 0$, and any $q, \tau \in \mathbb {R}$,

$$\begin{aligned} (q-p) g_{p, q}(\tau ) = \tau g_{p, q}'(\tau ) + q g_{p, q-2}(\tau ). \end{aligned}$$

### Proof

We proceed by induction on *p*. For $p=0$, by Definition 3.1, we have $(q-p) g_{p, q}(\tau ) = q$ while $\tau g_{p, q}'(\tau ) = 0$ and $q g_{p, q-2}(\tau ) = q$, so the claim is obviously true. Now let us prove the claim for $p \ge 1$, assuming that it is already true for all integer $0 \le p' \le p-1$. By Definition 3.1, we have

$$\begin{aligned} (q-p) g_{p, q}(\tau ) = (q-p) ( (1-\tau ^2) g_{p-1, q}'(\tau ) + (q-p+1) \tau g_{p-1, q}(\tau ) ). \end{aligned}$$

Rearranging, we obtain

$$\begin{aligned} (q-p) g_{p, q}(\tau )= & {} (q-p-1) \tau (q-p+1) g_{p-1, q}(\tau ) \\&+\, (1-\tau ^2) (q-p) g_{p-1, q}'(\tau ) + (q-p+1) \tau g_{p-1, q}(\tau ). \end{aligned}$$

By the induction hypothesis, applied for $p' := p-1$, we have

$$\begin{aligned} (q-p+1) g_{p-1, q}(\tau ) = \tau g_{p-1, q}'(\tau ) + q g_{p-1, q-2}(\tau ). \end{aligned}$$

for all $\tau \in \mathbb {R}$. Differentiating both sides, we obtain from this that

$$\begin{aligned} (q-p) g_{p-1, q}'(\tau ) = \tau g_{p-1, q}''(\tau ) + q g_{p-1, q-2}'(\tau ). \end{aligned}$$

Combining the above three formulas, we see that

6 $$\begin{aligned} (q-p) g_{p, q}(\tau )= & {} (q-p-1) \tau ( \tau g_{p-1, q}' + q g_{p-1, q-2}(\tau ) ) \nonumber \\&+\, (1-\tau ^2) ( \tau g_{p-1, q}''(\tau ) + q g_{p-1, q-2}'(\tau ) ) \nonumber \\&+\, (q-p+1) \tau g_{p-1, q}(\tau ). \end{aligned}$$

At the same time, by Lemma 4.1, we have

$$\begin{aligned} { \tau g_{p, q}'(\tau ) = (1-\tau ^2) \tau g_{p-1, q}''(\tau ) + (q-p-1) \tau ^2 g_{p-1, q}'(\tau ) + (q-p+1) \tau g_{p-1, q}(\tau ),} \end{aligned}$$

and, by Definition 3.1, we also have

$$\begin{aligned} q g_{p, q-2}(\tau ) = (1-\tau ^2) q g_{p-1, q-2}'(\tau ) + (q-p-1) \tau q g_{p-1, q-2}(\tau ). \end{aligned}$$

Summing the above two identities, we obtain the right-hand side of (6).$\square$

### Lemma 4.3

For any integer $p \ge 1$, and any $q, \tau \in \mathbb {R}$,

$$\begin{aligned} g_{p, q}'(\tau ) = (1-\tau ^2) g_{p-1, q}''(\tau ) + (q-p) \tau g_{p-1, q}'(\tau ) + q g_{p-1, q-2}(\tau ). \end{aligned}$$

### Proof

Apply Lemma 4.2 to the last term in (5).$\square$

The following lemma is particularly interesting. It turns out that, up to a constant factor, the derivative of the polynomial $g_{p, q}$ is exactly the previous polynomial but with a shifted value of *q*.

### Lemma 4.4

For any integer $p \ge 1$, and any $q \in \mathbb {R}$, we have $g_{p, q}' = p q g_{p-1, q-2}$.

### Proof

We proceed by induction on *p*. Let $\tau \in \mathbb {R}$. For $p=1$, we know from Definition 3.1 that $g_{p, q}(\tau ) = q \tau$, while $p q g_{p-1, q-2}(\tau ) = q$; therefore, the claim is indeed true. Now let us prove the claim for $p \ge 2$, assuming that it is already proved for all integer $0 \le p' \le p-1$. From Lemma 4.3, we already know that

$$\begin{aligned} g_{p, q}'(\tau ) = (1-\tau ^2) g_{p-1, q}''(\tau ) + (q-p) \tau g_{p-1, q}'(\tau ) + q g_{p-1, q-2}(\tau ). \end{aligned}$$

Therefore, it remains to prove that

$$\begin{aligned} (1-\tau ^2) g_{p-1, q}''(\tau ) + (q-p) \tau g_{p-1, q}'(\tau ) = (p-1) q g_{p-1, q-2}(\tau ). \end{aligned}$$

By the induction hypothesis for $p' := p-1$, we already have the identity $g_{p-1, q}' = (p-1) q g_{p-2, q-2}$ and in particular $g_{p-1, q}'' = (p-1) q {g_ {p-2, q-2}'}$. Thus,

$$\begin{aligned}&(1-\tau ^2) g_{p-1, q}''(\tau ) + (q-p) \tau g_{p-1, q}'(\tau ) \\&\quad = (p-1) q [ (1-\tau ^2) g_{p-2, q-2}'(\tau ) + (q-p) \tau g_{p-2, q-2}(\tau ) ]. \end{aligned}$$

It remains to verify that

$$\begin{aligned} (1-\tau ^2) g_{p-2, q-2}'(\tau ) + (q-p) \tau g_{p-2, q-2}(\tau ) = g_{p-1, q-2}(\tau ). \end{aligned}$$

But this is given directly by Definition 3.1.$\square$

Combined with Definition 3.1, Lemma 4.4 gives us a useful recursive formula for $g_{p, q}$ that does not involve any derivatives.

### Lemma 4.5

For any integer $p \ge 2$, and any $q, \tau \in \mathbb {R}$,

7 $$\begin{aligned} g_{p, q}(\tau ) = (1-\tau ^2) (p-1) q g_{p-2, q-2}(\tau ) + (q-p+1) \tau g_{p-1, q}(\tau ). \end{aligned}$$

Lemma 4.5 has several corollaries. The first one gives us closed-form expressions for the values of $g_{p, q}$ at the boundary points of the interval [0, 1].

### Proposition 4.2

For any integer $p \ge 0$, and any $q \in \mathbb {R}$, we have^1^

8 $$\begin{aligned} g_{p, q}(0) = {\left\{ \begin{array}{ll} (p-1)!! \prod _{i=0}^{\frac{p}{2}-1} (q - 2i), &{}\quad \mathrm{if}~p~\mathrm{even}, \\ 0, &{}\quad \mathrm{if}~p~\mathrm{odd}, \end{array}\right. } \end{aligned}$$

and

9 $$\begin{aligned} g_{p, q}(1) = \prod _{i=0}^{p-1} (q-i). \end{aligned}$$

### Proof

We proceed by induction on *p*. From Definition 3.1, we have $g_{0, q}(0) = g_{0, q}(1) = 1$ and $g_{1, q}(0) = 0$, $g_{1, q}(1) = q$. Thus, the claim is indeed true for $p=0$ and $p=1$. Now let us prove the claim for $p \ge 2$, assuming that it is already true for all integer $0 \le p' \le p-1$. Using Lemma 4.5, we obtain

10 $$\begin{aligned} g_{p, q}(0) = (p-1) q g_{p-2, q-2}(0). \end{aligned}$$

By the induction hypothesis, applied for $p' := p-2$ (and $q' := q-2$), we have

$$\begin{aligned} g_{p-2, q-2}(0) = {\left\{ \begin{array}{ll} (p-3)!! {\prod _{i=0}^{\frac{p}{2}-2}} (q-2-2i), &{}\quad \mathrm{if}~p~\mathrm{is~even}, \\ 0, &{}\quad \mathrm{if}~p~\mathrm{is~odd}. \end{array}\right. } \end{aligned}$$

By shifting the index in the product, this can be rewritten as

$$\begin{aligned} g_{p-2, q-2}(0) = {\left\{ \begin{array}{ll} (p-3)!! {\prod _{i=1}^{\frac{p}{2}-1}} (q-2i), &{}\quad \mathrm{if}~p~\mathrm{is~even}, \\ 0, &{}\quad \mathrm{if}~p~\mathrm{is~odd}. \end{array}\right. } \end{aligned}$$

Substituting this into (10), we obtain (8).

Similarly, by Lemma 4.5, we also have $g_{p, q}(1) = (q-p+1) g_{p-1, q}(1)$. But by the induction hypothesis, $g_{p-1, q}(1) = \prod _{i=0}^{p-2} (q-i)$, and we obtain (9).$\square$

The second corollary of Lemma 4.5 states that $g_{p, q}$ cannot take negative values on the interval [0, 1], provided that *q* is sufficiently large.

### Proposition 4.3

For any integer $p \ge 0$, and any real $q \ge p-1$, $g_{p, q}$ is nonnegative on [0, 1].

### Proof

We proceed by induction on *p*. Let $0 \le \tau \le 1$. For $p=0$, we know, by Definition 3.1, that $g_{p, q}(\tau ) = 1$, which is actually nonnegative for all real *q*. For $p=1$, by Definition 3.1, we have $g_{p, q}(\tau ) = q \tau$, which is indeed nonnegative when $q \ge p-1 = 0$.

Now let us prove the claim for $p \ge 2$, assuming that it is already proved for all integer $0 \le p' \le p-1$. From Lemma 4.5, we know that

$$\begin{aligned} g_{p, q}(\tau ) = (1-\tau ^2) (p-1) q g_{p-2, q-2}(\tau ) + (q-p+1) \tau g_{p-1, q}(\tau ). \end{aligned}$$

By the induction hypothesis, applied, respectively, for $p' := p-2$, $q' := q-2$ and $p' := p-1$, $q' := q$ (observe that in both cases $q' \ge p'-1$ since $q \ge p$), we have $g_{p-2, q-2}(\tau ) \ge 0$ and $g_{p-1, q}(\tau ) \ge 0$. Since $q \ge p-1 \ge 1$, then also $q-p+1 \ge 0$, and $(p-1) q \ge 0$. Thus, all parts in the right-hand side of the above formula are nonnegative.$\square$

Combining Proposition 4.3 with Lemma 4.4, we obtain that, when $q \ge p$, the polynomial $g_{p, q}$ is not only nonnegative but also monotonically increasing.

### Proposition 4.4

For any integer $p \ge 0$, and any real $q \ge p$, the derivative $g_{p, q}'$ is nonnegative on [0, 1]; hence $g_{p, q}$ is monotonically increasing on [0, 1].

Finally, let us show how we can apply the properties that we have established above, to find the maximal absolute value of $g_{p, q}$ on $[-1, 1]$.

### Proposition 4.5

For any integer $p \ge 0$, and any real $q \ge p$,

$$\begin{aligned} \max _{[-1, 1]} |g_{p, q}| = \prod _{i=0}^{p-1} (q-i). \end{aligned}$$

### Proof

By Proposition 4.1, we have $\max _{[-1, 1]} | g_{p, q} | = \max _{[0, 1]} |g_{p, q}|$. Since $g_{p, q}$ is nonnegative on [0, 1] (Proposition 4.3), $\max _{[0, 1]} |g_{p, q}| = \max _{[0, 1]} g_{p, q}$. By Proposition 4.4, $\max _{[0, 1]} g_{p, q} = g_{p, q}(1)$. But $g_{p, q}(1) = \prod _{i=0}^{p-1} (q-i)$ according to Proposition 4.2.$\square$

## Hölder Constants of Polynomials

We continue our study of polynomials $g_{p, q}$, but now we restrict our attention to the particular case when $q=p+\nu$ for some real $\nu \in [0, 1]$.

Clearly, the polynomial $g_{p, p+\nu }$ is $\nu$-Hölder continuous on $[-1, 1]$, since this is true for any other polynomial on a compact interval. The goal of this section is to obtain an explicit expression for the corresponding *Hölder constant*. We start with the result, allowing us to reduce our task to that on [0, 1].

### Theorem 5.1

For any integer $p \ge 0$, and any real $\nu \in [0, 1]$, the polynomial $g_{p, p+\nu }$ is $\nu$-Hölder continuous on $[-1, 1]$ with constant

$$\begin{aligned} \tilde{H}_{p, \nu } := {\left\{ \begin{array}{ll} H_{p, \nu }, &{}\quad \mathrm{if}~p~\mathrm{is~even}, \\ 2^{1-\nu } H_{p, \nu }, &{}\quad \mathrm{if}~p~\mathrm{is~odd}, \end{array}\right. } \end{aligned}$$

where $H_{p, \nu }$ is the corresponding Hölder constant of $g_{p, p+\nu }$ on [0, 1].

### Proof

Let $\tau _1, \tau _2 \in [-1, 1]$. We need to prove that

11 $$\begin{aligned} | g_{p, p+\nu }(\tau _2) - g_{p, p+\nu }(\tau _1) | \le \tilde{H}_{p, \nu } | \tau _2 - \tau _1 |^\nu . \end{aligned}$$

By Proposition 4.1, this inequality is invariant to negation transformations $(\tau _1, \tau _2) \mapsto (-\tau _1, -\tau _2)$. Therefore, we can assume that $\tau _2 \ge 0$. Furthermore, we can assume that $\tau _1 < 0$, since otherwise the claim is trivial.

**Case I** Suppose *p* is even. Then, by Proposition 4.1,

$$\begin{aligned} | g_{p, p+\nu }(\tau _2) - g_{p, p+\nu }(\tau _1) | = | g_{p, p+\nu }(\tau _2) - g_{p, p+\nu }(-\tau _1) |. \end{aligned}$$

Note that $-\tau _1, \tau _2 \in [0, 1]$. Therefore, by Hölder condition on [0, 1],

$$\begin{aligned} | g_{p, p+\nu }(\tau _2) - g_{p, p+\nu }(-\tau _1) | \le H_{p, \nu } |\tau _2 + \tau _1|^\nu . \end{aligned}$$

At the same time, $|\tau _2 + \tau _1| \le \tau _2 - \tau _1$ by the triangle inequality, and (11) follows.

**Case II** Now suppose *p* is odd. By Propositions 4.1 and 4.3,

$$\begin{aligned} | g_{p, p+\nu }(\tau _2) - g_{p, p+\nu }(\tau _1) | = g_{p, p+\nu }(\tau _2) + g_{p, p+\nu }(-\tau _1). \end{aligned}$$

Recall that $g_{p, p+\nu }(0) = 0$ (Proposition 4.2). Therefore,

$$\begin{aligned} g_{p, p+\nu }(\tau _2)= & {} g_{p, p+\nu }(\tau _2) - g_{p, p+\nu } (0) \le H_{p, \nu } \tau _2^\nu , \\ g_{p, p+\nu }(-\tau _1)= & {} g_{p, p+\nu }(-\tau _1) - g_{p, p+\nu }(0) \le H_{p, \nu } (-\tau _1)^\nu . \end{aligned}$$

Hence,

$$\begin{aligned} g_{p, p+\nu }(\tau _2) + g_{p, p+\nu }(-\tau _1) \le H_{p, \nu } ( \tau _2^\nu + (-\tau _1)^\nu ). \end{aligned}$$

To prove (11), it remains to show that $\tau _2^\nu + (-\tau _1)^\nu \le 2^ {1-\nu } (\tau _2 - \tau _1)^\nu$. But this follows from the concavity of power function $t \mapsto t^\nu$.$\square$

Our next task is to estimate the Hölder constant of $g_{p, p+\nu }$ on [0, 1]:

12 $$\begin{aligned} H_{p, \nu } := \max \limits _{0 \le \tau _1 < \tau _2 \le 1} \frac{ g_{p, p+\nu }(\tau _2) - g_{p, p+\nu }(\tau _1) }{ (\tau _2 - \tau _1)^\nu }. \end{aligned}$$

Note that Proposition 4.4 allows us to remove the absolute value sign.

### Theorem 5.2

For any integer $p \ge 0$, and any real $\nu \in [0, 1]$, we have

13 $$\begin{aligned} H_{p, \nu } \le \prod _{i=1}^p (\nu +i). \end{aligned}$$

The proof of Theorem 5.2 is based on two auxiliary propositions.

### Proposition 5.1

For any integer $p \ge 0$ and any real $\nu , \tau _1 \in [0, 1]$, the function

14 $$\begin{aligned} ]\tau _1, +\infty [ \rightarrow \mathbb {R}: \tau _2 \mapsto \frac{g_{p, p+\nu }(\tau _2) - g_{p, p+\nu }(\tau _1)}{(\tau _2 - \tau _1)^\nu } \end{aligned}$$

is monotonically increasing on $]\tau _1, 1]$.

### Proposition 5.2

For any integer $p \ge 0$ and any real $\nu \in [0, 1]$, the function

15 $$\begin{aligned} ]0, 1] \rightarrow \mathbb {R}: \tau \mapsto \frac{g_{p, p+\nu }(\tau )}{1 - (1-\tau )^\nu } \end{aligned}$$

is monotonically decreasing on ]0, 1].

Let us assume for a moment that these propositions are already proved. Then, the proof of Theorem 5.2 is simple.

### Proof

Let $0 \le \tau _1 < \tau _2 \le 1$. From Proposition 5.1, we know that

$$\begin{aligned} \frac{ g_{p, p+\nu }(\tau _2) - g_{p, p+\nu }(\tau _1) }{ (\tau _2 - \tau _1)^\nu } \le \frac{ g_{p, p+\nu }(1) - g_{p, p+\nu }(\tau _1) }{ (1-\tau _1)^\nu }. \end{aligned}$$

Therefore, to prove (13), it remains to show that

$$\begin{aligned} \frac{ g_{p, p+\nu }(1) - g_{p, p+\nu }(\tau _1) }{ (1-\tau _1)^\nu } \le \prod _{i=1}^p (\nu +i). \end{aligned}$$

Recall that, by Proposition 4.2, we have $\prod _{i=1}^p (\nu +i) = g_{p, p+\nu }(1)$. Thus, the inequality we need to prove is

$$\begin{aligned} \frac{ g_{p, p+\nu }(1) - g_{p, p+\nu }(\tau _1) }{ (1-\tau _1)^\nu } \le g_{p, p+\nu }(1), \end{aligned}$$

or, equivalently,

$$\begin{aligned} \frac{ g_{p, p+\nu }(\tau _1) }{ 1 - (1-\tau _1)^\nu } \ge g_{p, p+\nu }(1). \end{aligned}$$

But this follows from Proposition 5.2.

Our goal now is to prove Propositions 5.1 and 5.2.

We start with Proposition 5.1. It requires three technical lemmas.

### Lemma 5.1

For any integer $p \ge 0$, and any real $\nu , \tau \in [0, 1]$,

16 $$\begin{aligned} g_{p, p+\nu }(\tau ) \ge \tau g_{p, p+\nu }'(\tau ). \end{aligned}$$

Moreover, when $$p \ge 2$$,

17 $$\begin{aligned}&g_{p, p+\nu }(\tau ) - \tau g_{p, p+\nu }'(\tau ) \nonumber \\&\quad \ge (1-\tau ^2) (p-1) (p+\nu ) (g_{p-2, p-2+\nu }(\tau ) - \tau g_{p-2, p-2+\nu }'(\tau )). \end{aligned}$$

### Proof

First, let us prove (17). By Lemma 4.1, we have

$$\begin{aligned} g_{p, p+\nu }'(\tau ) = (1-\tau ^2) g_{p-1, p+\nu }''(\tau ) + (\nu -1) \tau g_{p-1, p+\nu }'(\tau ) + (\nu +1) g_{p-1, p+\nu }(\tau ). \end{aligned}$$

Since $g_{p-1, p+\nu }'(\tau ) \ge 0$ (Proposition 4.4) and $\nu \le 1$, it follows that

$$\begin{aligned} g_{p, p+\nu }'(\tau ) \le (1-\tau ^2) g_{p-1, p+\nu }''(\tau ) + (\nu +1) g_{p-1, p+\nu }(\tau ). \end{aligned}$$

At the same time, by Definition 3.1,

$$\begin{aligned} g_{p, p+\nu }(\tau ) = (1-\tau ^2) g_{p-1, p+\nu }'(\tau ) + {(\nu +1) \tau g_{p-1, p+\nu }(\tau )}. \end{aligned}$$

Thus,

$$\begin{aligned} g_{p, p+\nu }(\tau ) - \tau g_{p, p+\nu }'(\tau ) \ge (1-\tau ^2) (g_{p-1, p+\nu }'(\tau ) - \tau g_{p-1, p+\nu }''(\tau )). \end{aligned}$$

Applying Lemma 4.4, we obtain that

$$\begin{aligned} g_{p-1, p+\nu }'(\tau )= & {} (p-1) (p+\nu ) g_{p-2, p-2+\nu }(\tau ), \\ g_{p-1, p+\nu }''(\tau )= & {} (p-1) (p+\nu ) g_{p-2, p-2+\nu }'(\tau ), \end{aligned}$$

and (17) follows.

It remains to prove (16). For $p=0$, we have $g_{p, p+\nu }(\tau ) = 1$ (Definition 3.1), and hence, $\tau g_{p, p+\nu }'(\tau ) = 0$, and (16) is indeed true. For $p=1$, by Definition 3.1, we have $g_{p, p+\nu }(\tau ) = (p+\nu ) \tau$, and hence, $\tau g_{p, p+\nu }'(\tau ) = (p+\nu ) \tau$, and (16) is again true. The general case $p \ge 2$ easily follows from (17) by induction.$\square$

### Lemma 5.2

For any integer $p \ge 0$, any real $\nu \in [0, 1]$, and $0 \le \tau _1 \le \tau _2 \le 1$,

18 $$\begin{aligned} (p+\nu ) g_{p, p-2+\nu }(\tau _2) \le \nu (g_{p, p+\nu }(\tau _1) - \tau _1 g_{p, p+\nu }'(\tau _1)). \end{aligned}$$

### Proof

We use induction in *p*. For $p=0$, we have $g_{p, p-2+\nu }(\tau _2) = 1$, while $g_{p, p+\nu }(\tau _1) - \tau _1 g_{p, p+\nu }'(\tau _1) = 1$ (see Definition 3.1), so the claim is true. For $p=1$, we have $g_{p, p-2+\nu }(\tau _2) = -(1-\nu ) \tau _2 \le 0$ while $g_{p, p+\nu }(\tau _1) - \tau _1 g_{p, p+\nu }'(\tau _1) = 0$, (see Definition 3.1), and hence, the claim is again true.

Now we prove the claim for $p \ge 2$, assuming that it is already true for all integer $0 \le p' \le p-1$. According to Lemma 4.5, we have

$$\begin{aligned} g_{p, p-2+\nu }(\tau _2)= & {} (1-\tau _2^2) (p-1) (p-2+\nu ) g_{p-2, p-4+\nu }(\tau _2) \\&-\,(1-\nu ) \tau _2 g_{p-1, p-2+\nu }(\tau _2). \end{aligned}$$

Since $g_{p-1, p-2+\nu }(\tau _2) \ge 0$ (Proposition 4.3), we further have

$$\begin{aligned} g_{p, p-2+\nu }(\tau _2) \le (1-\tau _2^2) (p-1) (p-2+\nu ) g_{p-2, p-4+\nu }(\tau _2). \end{aligned}$$

If $g_{p-2, p-4+\nu }(\tau _2) \le 0$, it follows that $g_{p, p-2+\nu }(\tau _2) \le 0$, and the proof in this case is finished, because the right-hand side in (18) is always nonnegative in view of Lemma 5.1. Therefore, we can assume that $g_{p-2, p-4+\nu }(\tau _2) \ge 0$.

Since $$\tau _2 \ge \tau _1$$, then

$$\begin{aligned} g_{p, p-2+\nu }(\tau _2) \le (1-\tau _1^2) (p-1) (p-2+\nu ) g_{p-2, p-4+\nu }(\tau _2). \end{aligned}$$

Applying the inductive assumption to $p' := p-2$, we obtain

$$\begin{aligned} (p-2+\nu ) g_{p-2, p-4+\nu }(\tau _2) \le \nu (g_{p-2, p-2+\nu }(\tau _1) - \tau _1 g_{p-2, p-2+\nu }'(\tau _1)). \end{aligned}$$

Hence,

$$\begin{aligned} g_{p, p-2+\nu }(\tau _2) \le \nu (1-\tau _1^2) (p-1) (g_{p-2, p-2+\nu }(\tau _1) - \tau _1 g_{p-2, p-2+\nu }'(\tau _1)). \end{aligned}$$

Thus, to finish the proof, it remains to show that

$$\begin{aligned}&(1-\tau _1^2) (p-1) (p+\nu ) (g_{p-2, p-2+\nu }(\tau _1) - \tau _1 g_{p-2, p-2+\nu }'(\tau _1)) \\&\quad \le g_{p, p+\nu }(\tau _1) - \tau _1 g_{p, p+\nu }'(\tau _1). \end{aligned}$$

But this is guaranteed by Lemma 5.1.$\square$

### Lemma 5.3

For any integer $p \ge 0$, and any real $\nu , \tau _2 \in [0, 1]$, the function

19 $$\begin{aligned} ]0, +\infty [ \rightarrow \mathbb {R}: \tau _1 \mapsto \frac{ \nu g_{p, p+\nu }(\tau _1) - (p+\nu ) g_{p, p-2+\nu }(\tau _2) }{\tau _1} \end{aligned}$$

is monotonically decreasing on $]0, \tau _2]$.

### Proof

The function (19) is differentiable with derivative

$$\begin{aligned} \frac{ \nu (\tau _1 g_{p, p+\nu }'(\tau _1) - g_{p, p+\nu }(\tau _1)) + (p+\nu ) g_{p, p-2+\nu }(\tau _2) }{ \tau _1^2 }, \end{aligned}$$

which is non-positive on $]0, \tau _2]$ by Lemma 5.2.$\square$

Now we can present the proof of Proposition 5.1:

### Proof

Since (14) is differentiable, it suffices to prove that its derivative

$$\begin{aligned} \frac{g_{p, p+\nu }'(\tau _2)}{(\tau _2 - \tau _1)^\nu } - \frac{\nu (g_{p, p+\nu }(\tau _2) - g_{p, p+\nu }(\tau _1))}{(\tau _2 - \tau _1)^{\nu +1}} \end{aligned}$$

is nonnegative for all $0< \tau _1 < \tau _2 \le 1$, or, equivalently, that

$$\begin{aligned} g_{p, p+\nu }'(\tau _2) (\tau _2 - \tau _1) \ge \nu (g_{p, p+\nu }(\tau _2) - g_{p, p+\nu }(\tau _1)). \end{aligned}$$

By Lemma 4.2,

20 $$\begin{aligned} \nu g_{p, p+\nu }(\tau _2) = \tau _2 g_{p, p+\nu }'(\tau _2) + (p+\nu ) g_{p, p-2+\nu }(\tau _2). \end{aligned}$$

Therefore, it is enough to prove that

$$\begin{aligned} \nu g_{p, p+\nu }(\tau _1) - (p+\nu ) g_{p, p-2+\nu }(\tau _2) \ge \tau _1 g_{p, p+\nu }'(\tau _2), \end{aligned}$$

or, equivalently,

21 $$\begin{aligned} \frac{ \nu g_{p, p+\nu }(\tau _1) - (p+\nu ) g_{p, p-2+\nu }(\tau _2) }{ \tau _1 } \ge g_{p, p+\nu }'(\tau _2). \end{aligned}$$

But this immediately follows from Lemma 5.3 using (20).$\square$

It remains to prove Proposition 5.2. For this, we need one more lemma.

### Lemma 5.4

For any integer $p \ge 0$, and any real $\nu , \tau \in [0, 1]$, we have

22 $$\begin{aligned} (p+\nu ) g_{p, p-2+\nu }(\tau ) \ge -(1 - (1-\tau )^{1-\nu }) g_{p, p+\nu }'(\tau ). \end{aligned}$$

### Proof

As usual, we use induction in *p*. The base case $p=0$ is trivial, since $g_{p, p-2+\nu }(\tau ) = 1$, while $g_{p, p+\nu }'(\tau ) = 0$ (see Definition 3.1). To prove the general case $p \ge 1$, we assume that (22) is already true for all integer $0 \le p' \le p-1$.

Our first step is to show that

23 $$\begin{aligned} (p+\nu ) g_{p, p-2+\nu }(\tau )\ge & {} -(1-\tau ^2) (1 - (1-\tau )^{1-\nu }) g_{p-1, p+\nu }''(\tau ) \nonumber \\&-\,(p+\nu ) (1-\nu ) \tau g_{p-1, p-2+\nu }(\tau ). \end{aligned}$$

If $p=1$, we have $g_{p, p-2+\nu }(\tau ) = -(1-\nu ) \tau$, while $g_{p-1, p+\nu }''(\tau ) = 0$ and $g_{p-1, p-2+\nu }(\tau ) = 1$ (see Definition 3.1), so (23) is indeed true. To justify it for all other $p \ge 2$, we proceed as follows. By Lemma 4.5, we know that

$$\begin{aligned} g_{p, p-2+\nu }(\tau )= & {} (1-\tau ^2) (p-1) (p-2+\nu ) g_{p-2, p-4+\nu }(\tau ) \\&- (1-\nu ) \tau g_{p-1, p-2+\nu }(\tau ). \end{aligned}$$

Therefore, (23) is equivalent to

$$\begin{aligned} (p+\nu ) (p-1) (p-2+\nu ) g_{p-2, p-4+\nu }(\tau ) \ge -(1 - (1-\tau )^{1-\nu }) g_{p-1, p+\nu }''(\tau ). \end{aligned}$$

By our inductive assumption (22), applied to $p' := p-2$, we already have

$$\begin{aligned} (p-2+\nu ) g_{p-2, p-4+\nu }(\tau ) \ge -(1 - (1-\tau )^{1-\nu }) g_{p-2, p-2+\nu }'(\tau ). \end{aligned}$$

At the same time, by Lemma 4.4,

$$\begin{aligned} (p+\nu ) (p-1) g_{p-2, p-2+\nu }'(\tau ) = g_{p-1, p+\nu }''(\tau ). \end{aligned}$$

Thus, (23) is established.

Now we estimate the right-hand side in (23). Applying Lemma 4.3 and the fact that $g_{p-1, p+\nu }'(\tau ) \ge 0$ (Proposition 4.4), we obtain

$$\begin{aligned} g_{p, p+\nu }'(\tau )= & {} (1-\tau ^2) g_{p-1, p+\nu }''(\tau ) + \nu \tau g_{p-1, p+\nu }'(\tau ) + (p+\nu ) g_{p-1, p-2+\nu }(\tau ) \\\ge & {} (1-\tau ^2) g_{p-1, p+\nu }''(\tau ) + (p+\nu ) g_{p-1, p-2+\nu }(\tau ). \end{aligned}$$

From this, it follows that

$$\begin{aligned} (1-\tau ^2) g_{p-1, p+\nu }''(\tau ) \le g_{p, p+\nu }'(\tau ) - (p+\nu ) g_{p-1, p-2+\nu }(\tau ). \end{aligned}$$

Substituting the above equation into (23), we obtain

$$\begin{aligned} (p+\nu ) g_{p, p-2+\nu }(\tau )\ge & {} -(1 - (1-\tau )^{1-\nu }) g_{p, p+\nu }'(\tau ) \\&+\,(p+\nu ) (1 - (1-\nu ) \tau - (1-\tau )^{1-\nu }) g_{p-1, p-2+\nu }(\tau ). \end{aligned}$$

Since $g_{p-1, p-2+\nu }(\tau ) \ge 0$ (by Proposition 4.3), it only remains to show that $(1-\tau )^{1-\nu } \le 1 - (1-\nu ) \tau$. But this follows from the concavity of power function $\tau \mapsto (1 - \tau )^{1-\nu }$.$\square$

Now we can give the proof of Proposition 5.2:

### Proof

Since (15) is differentiable, it suffices to prove that its derivative

$$\begin{aligned} \frac{ ( 1 - (1-\tau )^\nu ) g_{p, p+\nu }'(\tau ) - \nu (1-\tau )^{\nu -1} g_{p, p+\nu }(\tau ) }{ (1 - (1-\tau )^\nu )^2 } \end{aligned}$$

is non-positive for all $0< \tau < 1$. By Lemma 4.2, we have

$$\begin{aligned} \nu g_{p, p+\nu }(\tau ) = \tau g_{p, p+\nu }'(\tau ) + (p+\nu ) g_{p, p-2+\nu }(\tau ). \end{aligned}$$

Thus, we need to show that

$$\begin{aligned} (1-\tau )^{\nu -1} \left( \tau g_{p, p+\nu }'(\tau ) + (p+\nu ) g_{p, p-2+\nu } (\tau ) \right) \ge (1 - (1-\tau )^\nu ) g_ {p, p+\nu }'(\tau ), \end{aligned}$$

or, equivalently (by multiplying both sides by $(1-\tau )^ {1-\nu }$), that

$$\begin{aligned} \tau g_{p, p+\nu }'(\tau ) + (p+\nu ) g_{p, p-2+\nu } (\tau ) \ge ((1-\tau )^{1-\nu } - 1+\tau ) g_{p, p+\nu }'(\tau ), \end{aligned}$$

or, equivalently (by moving the first term into the right-hand side), that

$$\begin{aligned} (p+\nu ) g_{p, p-2+\nu }(\tau ) \ge -(1 - (1-\tau )^{1-\nu }) g_{p, p+\nu }'(\tau ). \end{aligned}$$

But this is given by Lemma 5.4.$\square$

To conclude this section, let us discuss the optimality of Theorem 5.2.

For odd values of *p*, the obtained constant $\prod _ {i=1}^p (\nu +i)$ turns out to be optimal. Indeed, using $\tau _1 := 0$, $\tau _2 := 1$ in (12) and taking into account Proposition 4.2, we obtain that

$$\begin{aligned} H_{p, \nu } \ge \frac{g_{p, p+\nu }(\tau _2) - g_{p, p+\nu }(\tau _1)}{(\tau _2 - \tau _1)^\nu } = g_{p, p+\nu }(1) = \prod _{i=1}^p (\nu +i). \end{aligned}$$

However, for even *p*, this constant is suboptimal. For example, consider the case when $p=2$. We know that

$$\begin{aligned} g_{2, 2+\nu }(\tau ) = (\nu +2) (\nu \tau ^2 + 1). \end{aligned}$$

The corresponding optimal constant, according to Proposition 5.1, is

$$\begin{aligned} H_{2, \nu } = \max \limits _{0 \le \tau< 1} \frac{ g_{2, 2+\nu }(1) - g_{2, 2+\nu }(\tau ) }{ (1-\tau )^\nu } = \nu (\nu +2) \max \limits _{0 \le \tau < 1} (1-\tau )^{1-\nu } (1+\tau ). \end{aligned}$$

Note that this maximization problem is logarithmically concave in $\tau$. Taking the logarithm and setting the derivative to zero, we find that the maximal point corresponds to $\tau := \frac{\nu }{2-\nu } \in [0, 1]$, and the corresponding optimal value is

$$\begin{aligned} H_{2, \nu } = \nu (\nu +2) \frac{2^{2-\nu } (1-\nu )^{1-\nu }}{(2-\nu )^{2-\nu }} \le (\nu +1) (\nu +2). \end{aligned}$$

Of course, the last inequality is strict for all $0 \le \nu < 1$.

## Hölder Continuity of Derivatives of Powers of Euclidean Norm

We have established the main properties of polynomials $g_{p, q}$ and obtained an explicit upper bound on their Hölder constant. Hence, we are ready to prove the Hölder continuity of derivatives of powers of Euclidean norm. Let us start with a simple result that gives us a lower bound on the Hölder constant.

### Theorem 6.1

For any integer $p \ge 0$, and any real $\nu \in [0, 1]$, the Hölder constant of $D^p f_{p+\nu }$, corresponding to degree $\nu$, cannot be smaller than

24 $$\begin{aligned} C_{p, \nu } := {\left\{ \begin{array}{ll} \prod _{i=1}^p (\nu +i), &{}\quad \mathrm{if}~p~\mathrm{is~even}, \\ 2^{1-\nu } \prod _{i=1}^p (\nu +i), &{}\quad \mathrm{if}~p~\mathrm{is~odd}. \end{array}\right. } \end{aligned}$$

### Proof

According to (1), we need to show that

$$\begin{aligned} | D^p f_{p+\nu }(x_2)[h]^p - D^p f_{p+\nu }(x_1)[h]^p | \ge C_{p, \nu } \Vert x_2 - x_1 \Vert ^\nu \end{aligned}$$

for some $x_1, x_2 \in \mathbb {E}$ and some unit $h \in \mathbb {E}$. Let us choose an arbitrary unit vector $h \in \mathbb {E}$, and set $x_2 := h$. By Theorem 3.1 and Proposition 4.2,

$$\begin{aligned} D^p f_{p+\nu }(x_2)[h]^p = \Vert x_2 \Vert ^\nu g_{p, p+\nu }(1) = \prod _{i=1}^p (\nu +i). \end{aligned}$$

To specify $x_1$, we consider two cases.

If *p* is even, set $x_1 := 0$. Then, $D^p f_{p+\nu }(x_1)[h]^p = 0$ by Theorem 3.1, and

$$\begin{aligned} | D^p f_{p+\nu }(x_2)[h]^p - D^p f_{p+\nu }(x_1)[h]^p | = \prod _{i=1}^p (\nu +i), \end{aligned}$$

which is exactly $C_{p, \nu } \Vert x_2 - x_1 \Vert ^\nu$. If *p* is odd, we take $x_1 := -h$. This gives us

$$\begin{aligned} D^p f_{p+\nu }(x_1)[h]^p = \Vert x_1 \Vert ^\nu g_{p, p+\nu }(-1) = -\prod _{i=1}^p (\nu +i), \end{aligned}$$

where we apply Proposition 4.1 to rewrite $g_{p, p+\nu }(-1) = g_{p, p+\nu }(1)$. Hence,

$$\begin{aligned} | D^p f_{p+\nu }(x_2)[h]^p - D^p f_{p+\nu }(x_1)[h]^p | = 2 \prod _{i=1}^p (\nu +i), \end{aligned}$$

which is again precisely $C_{p, \nu } \Vert x_2 - x_1 \Vert ^\nu$.$\square$

Next we prove Hölder continuity with the optimal constant along any line, passing through the origin.

### Theorem 6.2

For any integer $p \ge 0$, and any real $\nu \in [0, 1]$, the restriction of $D^p f_{p+\nu }$ to a line, passing through the origin, is $\nu$-Hölder continuous with constant $C_{p, \nu }$.

### Proof

Let $x_1, x_2 \in \mathbb {E}$ be arbitrary points, lying on a line, passing through the origin, and let $h \in \mathbb {E}$ be an arbitrary unit vector. According to (1) and Theorem 3.1, we need to show that

$$\begin{aligned} | \Vert x_2 \Vert ^\nu g_{p, p+\nu }(\tau _2) - \Vert x_1 \Vert ^\nu g_{p, p+\nu }(\tau _1) | \le C_{p, \nu } \Vert x_2 - x_1 \Vert ^\nu , \end{aligned}$$

where $$\tau _1 := \tau _h(x_1)$$, $$\tau _2 := \tau _h(x_2)$$.

Observe that this inequality is symmetric in $x_1$ and $x_2$ and is invariant when we replace the pair $(x_1, x_2)$ with $(-x_1, -x_2)$. Therefore, we can assume that $\Vert x_2 \Vert \ge \Vert x_1 \Vert$ and $\tau _2 \ge 0$.

Since $x_1$ and $x_2$ lie on a line, passing through the origin, $\tau _1$ and $\tau _2$ can differ only in sign. Hence, by Proposition 4.1, we have two options: either $g_{p, p+\nu }(\tau _1) = g_{p, p+\nu }(\tau _2)$ or $g_{p, p+\nu }(\tau _1) = -g_{p, p+\nu }(\tau _2)$.

**Case I** Suppose $g_{p, p+\nu }(\tau _1) = g_{p, p+\nu }(\tau _2)$ (while $\tau _1$ can be of any sign). Then,

$$\begin{aligned} | \Vert x_2 \Vert ^\nu g_{p, p+\nu }(\tau _2) - \Vert x_1 \Vert ^\nu g_{p, p+\nu }(\tau _1) | = |g_{p, p+\nu }(\tau _2)| (\Vert x_2 \Vert ^\nu - \Vert x_1 \Vert ^\nu ). \end{aligned}$$

By Proposition 4.5 and (24), we know that

25 $$\begin{aligned} |g_{p, p+\nu }(\tau _2)| \le \prod _{i=1}^p (\nu +i) \le C_{p, \nu }. \end{aligned}$$

Thus, it suffices to prove that $\Vert x_2 \Vert ^\nu - \Vert x_1 \Vert ^\nu \le \Vert x_2 - x_1 \Vert ^\nu$. But this follows from the well-known inequality $r_2^\nu - r_1^\nu \le (r_2 - r_1)^\nu$ (which is valid for any real $0 \le r_1 \le r_2$) combined with the reverse triangle inequality.

**Case II** Suppose $g_{p, p+\nu } (\tau _1) = -g_{p, p+\nu }(\tau _2)$ ($\ne 0$). By Proposition 4.1 and Proposition 4.3, this happens only if *p* is odd and $\tau _1 \le 0$. Thus, $\tau _1 = -\tau _2$, and

26 $$\begin{aligned} | \Vert x_2 \Vert ^\nu g_{p, p+\nu }(\tau _2) - \Vert x_1 \Vert ^\nu g_{p, p+\nu }(\tau _1) | = |g_{p, p+\nu }(\tau _2)| (\Vert x_2 \Vert ^\nu + \Vert x_1 \Vert ^\nu ). \end{aligned}$$

Due to (25), it remains to prove that $$\Vert x_2 \Vert ^\nu + \Vert x_1 \Vert ^\nu \le 2^{1-\nu } \Vert x_2 - x_1 \Vert ^\nu $$. But this is immediate. Indeed, $$\Vert x_2 \Vert ^\nu + \Vert x_1 \Vert ^\nu \le 2^{1-\nu } (\Vert x_2 \Vert + \Vert x_1 \Vert )^\nu $$ by the concavity of the power function $$t \mapsto t^\nu $$, while $$\Vert x_2 \Vert + \Vert x_1 \Vert = \Vert x_2 - x_1 \Vert $$ since the segment $$ [x_1, x_2]$$ contains the origin..$$\square $$

Our final step is to extend Hölder continuity from lines, passing through the origin, onto the whole space. The main instrument for doing this is exploiting Hölder continuity of $g_{p, p+\nu }$ that we studied in Sect. 5.

### Theorem 6.3

For any integer $p \ge 0$, and any real $\nu \in [0, 1]$, $D^p f_{p+\nu }$ is $\nu$-Hölder continuous with constant

27 $$\begin{aligned} A_{p, \nu } := {\left\{ \begin{array}{ll} (p-1)!! \prod _{i=1}^{p/2} (\nu + 2i) + H_{p, \nu }, &{}\quad \mathrm{if}~p~\mathrm{is~even}, \\ 2^{1-\nu } \prod _{i=1}^p (\nu + i), &{}\quad \mathrm{if}~p~\mathrm{is~odd}, \end{array}\right. } \end{aligned}$$

where $H_{p, \nu }$ is the constant of $\nu$-Hölder continuity of $g_{p, p+\nu }$ on [0, 1]. In particular, $D^p f_{p+\nu }$ is $\nu$-Hölder continuous with constant

$$\begin{aligned} \tilde{A}_{p, \nu } := {\left\{ \begin{array}{ll} (p-1)!! \prod _{i=1}^{p/2} (\nu + 2i) + \prod _{i=1}^p (\nu +i), &{}\quad \mathrm{if}~p~\mathrm{is~even}, \\ 2^{1-\nu } \prod _{i=1}^p (\nu +i), &{}\quad \mathrm{if}~p~\mathrm{is~odd}. \end{array}\right. } \end{aligned}$$

### Proof

First of all, observe that the constant $A_{p, \nu }$ is not smaller than the corresponding lower bound $C_{p, \nu }$ given by Theorem 6.1:

28 $$\begin{aligned} C_{p, \nu } \le A_{p, \nu }. \end{aligned}$$

Indeed, for odd values of *p*, these constants coincide. When *p* is even, (28) follows from the following trivial lower bound for the Hölder constant $H_{p, \nu }$:

$$\begin{aligned} H_{p, \nu } \ge g_{p, p+\nu }(1) - g_{p, p+\nu }(0) = \prod _{i=1}^p (\nu +i) - (p-1)!! \prod _{i=1}^{p/2} (\nu +2i), \end{aligned}$$

where the last equality is due to Proposition 4.2.

Second, observe that we only need to prove the first claim, since the other one follows directly from the first one and Theorem 5.2.

Let $x_1, x_2 \in \mathbb {E}$ and let $h \in \mathbb {E}$ be an arbitrary unit vector. In view of (1) and Theorem 3.1, we need to show that

29 $$\begin{aligned} | \Vert x_2 \Vert ^\nu g_{p, p+\nu }(\tau _2) - \Vert x_1 \Vert ^\nu g_{p, p+\nu }(\tau _1) | \le A_{p, \nu } \Vert x_2 - x_1 \Vert ^\nu , \end{aligned}$$

where $$\tau _1 := \tau _h(x_1)$$, $$\tau _2 := \tau _h(x_2)$$.

Due to invariance of the above inequality to transformations of the form $(x_1, x_2) \mapsto (x_2, x_1)$ and $(x_1, x_2) \mapsto (-x_1, -x_2)$, we can assume in what follows that $\Vert x_1 \Vert \le \Vert x_2 \Vert$ and $\tau _2 \ge 0$. Furthermore, we can also assume that $x_1 \ne 0$ (and hence $x_2 \ne 0$), since otherwise the claim follows from Theorem 6.2.

There are now several cases to consider.

**Case I** Suppose $g_{p, p+\nu } (\tau _1) < 0$. By Propositions 4.1 and 4.3, this happens only if *p* is odd and $\tau _1 \le 0$. Then, $g_{p, p+\nu }(\tau _1) = -g_{p, p+\nu }(-\tau _1)$, and

$$\begin{aligned} | \Vert x_2 \Vert ^\nu g_{p, p+\nu }(\tau _2) - \Vert x_1 \Vert ^\nu g_{p, p+\nu }(\tau _1) | = \Vert x_2 \Vert ^\nu g_{p, p+\nu }(\tau _2) + \Vert x_1 \Vert ^\nu g_{p, p+\nu }(-\tau _1), \end{aligned}$$

where we have removed the absolute value sign, because all terms in the right-hand side are nonnegative (see Proposition 4.3).

Since *p* is odd, $g_{p, p+\nu }(0) = 0$ (see Proposition 4.1). Therefore, by the definition of $H_{p, \nu }$, it follows that

$$\begin{aligned} g_{p, p+\nu }(-\tau _1)= & {} g_{p, p+\nu }(-\tau _1) - g_{p, p+\nu }(0) \le H_{p, \nu } (-\tau _1^\nu ), \\ g_{p, p+\nu }(\tau _2)= & {} g_{p, p+\nu }(\tau _2) - g_{p, p+\nu }(0) \le H_{p, \nu } \tau _2^\nu . \end{aligned}$$

Combining this with the concavity of power function $t \mapsto t^\nu$, we obtain

$$\begin{aligned} \Vert x_2 \Vert ^\nu g_{p, p+\nu }(\tau _2) + \Vert x_1 \Vert ^\nu g_{p, p+\nu }(-\tau _1)\le & {} H_{p, \nu } ( (\Vert x_2 \Vert \tau _2)^\nu + (-\Vert x_1 \Vert \tau _1)^\nu ) \\\le & {} 2^{1-\nu } H_{p, \nu } ( \Vert x_2 \Vert \tau _2 - \Vert x_1 \Vert \tau _1 )^\nu . \end{aligned}$$

Note that $2^{1-\nu } H_{p, \nu } \le A_{p, \nu }$ by Theorem 5.2. Thus, it remains to show that $\Vert x_2 \Vert \tau _2 - \Vert x_1 \Vert \tau _1 \le \Vert x_2 - x_1 \Vert$. But this follows from the Cauchy–Schwartz inequality since $\Vert x_2 \Vert \tau _2 - \Vert x_1 \Vert \tau _1 = \langle B (x_2 - x_1), h \rangle$ by the definition of $\tau _1$, $\tau _2$.

**Case II** Now suppose $g_{p, p+\nu }(\tau _1) \ge 0$ (while $\tau _1$ can have any sign). We prove (29) by proving separately two inequalities with the removed absolute value sign.

First, we show that

30 $$\begin{aligned} \Vert x_1 \Vert ^\nu g_{p, p+\nu }(\tau _1) - \Vert x_2 \Vert ^\nu g_{p, p+\nu }(\tau _2) \le A_{p, \nu } \Vert x_2 - x_1 \Vert ^\nu . \end{aligned}$$

Let $x_2':= \frac{\Vert x_1 \Vert }{\Vert x_2 \Vert } x_2$ be the radial projection of $x_2$ onto the sphere with radius $r := \Vert x_1 \Vert$, centered at the origin. Note that

31 $$\begin{aligned} \tau _2' := \tau _h(x_2') = \tau _2, \qquad \Vert x_2' \Vert = r \le \Vert x_2 \Vert , \quad \Vert x_2' - x_1 \Vert \le \Vert x_2 - x_1 \Vert . \end{aligned}$$

The first two relations are evident. The last one follows from the fact that projections onto convex sets decrease distances and can be explicitly proved as follows. First, by the Cauchy–Schwartz inequality, we have $\langle B x_1, x_2 \rangle \le \rho \Vert x_2 \Vert ^2$, where $\rho := \frac{\Vert x_1 \Vert }{\Vert x_2 \Vert } \le 1$. Therefore, using the fact that $x_2' = \rho x_2$, we obtain

$$\begin{aligned} \begin{aligned} \Vert x_2&- x_1 \Vert ^2 - \Vert x_2' - x_1 \Vert ^2 = \Vert x_2 \Vert ^2 - \Vert x_2' \Vert ^2 - 2 \langle B x_1, x_2 - x_2' \rangle \\&= (1 - \rho ^2) \Vert x_2 \Vert ^2 - 2 (1 - \rho ) \langle B x_1, x_2 \rangle \ge (1 - \rho ^2) \Vert x_2 \Vert ^2 - 2 (1 - \rho ) \rho \Vert x_2 \Vert ^2 \\&= (1 - \rho )^2 \Vert x_2 \Vert ^2 \ge 0. \end{aligned} \end{aligned}$$

Since $g_{p, p+\nu }(\tau _2) \ge 0$ (Proposition 4.3), from (31) it follows that

$$\begin{aligned} \Vert x_1 \Vert ^\nu g_{p, p+\nu }(\tau _1) - \Vert x_2 \Vert ^\nu g_{p, p+\nu }(\tau _2) \le r^\nu (g_{p, p+\nu }(\tau _1) - g_{p, p+\nu }(\tau _2')). \end{aligned}$$

At the same time, by Theorem 5.1,

$$\begin{aligned} g_{p, p+\nu }(\tau _1) - g_{p, p+\nu }(\tau _2') \le \tilde{H}_{p, \nu } |\tau _2' - \tau _1|^\nu . \end{aligned}$$

Hence,

32 $$\begin{aligned} \Vert x_1 \Vert ^\nu g_{p, p+\nu }(\tau _1) - \Vert x_2 \Vert ^\nu g_{p, p+\nu }(\tau _2) \le \tilde{H}_{p, \nu } (r |\tau _2' - \tau _1|)^\nu . \end{aligned}$$

Note that

$$\begin{aligned} r |\tau _2' - \tau _1| = | \Vert x_2' \Vert \tau _2' - \Vert x_1 \Vert \tau _1 | = | \langle B (x_2' - x_1), h \rangle |. \end{aligned}$$

Therefore, by Cauchy–Schwartz inequality and (31), we have

$$\begin{aligned} r |\tau _2' - \tau _1| \le \Vert x_2' - x_1 \Vert \le \Vert x_2 - x_1 \Vert . \end{aligned}$$

Substituting this into (32), we obtain

$$\begin{aligned} \Vert x_1 \Vert ^\nu g_{p, p+\nu }(\tau _1) - \Vert x_2 \Vert ^\nu g_{p, p+\nu }(\tau _2) \le \tilde{H}_{p, \nu } \Vert x_2 - x_1 \Vert ^\nu . \end{aligned}$$

This finishes the proof of (30), because $\tilde{H}_{p, \nu } \le A_{p, \nu }$ by Theorem 5.2.

It remains to show the reverse inequality

33 $$\begin{aligned} \Vert x_2 \Vert ^\nu g_{p, p+\nu }(\tau _2) - \Vert x_1 \Vert ^\nu g_{p, p+\nu }(\tau _1) \le A_{p, \nu } \Vert x_2 - x_1 \Vert ^\nu . \end{aligned}$$

For this, we consider two subcases.

**Case II(a)** Suppose $\tau _1 \ge \tau _2$. Let $x_1' := \frac{\langle B x_1, x_2 \rangle }{ \Vert x_2 \Vert ^2 } x_2$ be the projection of $x_1$ onto the line, connecting $x_2$ with the origin, and let $\tau _1':= \tau _h (x_1')$. Then,

34 $$\begin{aligned} \Vert x_1' \Vert \le \Vert x_1 \Vert , \qquad \Vert x_2 - x_1' \Vert \le \Vert x_2 - x_1 \Vert . \end{aligned}$$

Furthermore,

35 $$\begin{aligned} g_{p, p+\nu }(\tau _1') \le g_{p, p+\nu }(\tau _2). \end{aligned}$$

Indeed, if $\langle B x_1, x_2 \rangle \ge 0$, then $\tau _1' = \tau _2$ and $g_{p, p+\nu }(\tau _1') = g_{p, p+\nu }(\tau _2)$; otherwise, $\tau _1' = -\tau _2$, and hence, $g_{p, p+\nu }(\tau _1') = (-1)^p g_{p, p+\nu }(\tau _2)$ (Proposition 4.1), which either coincides with $g_{p, p+\nu }(\tau _2)$ when *p* is even, or becomes $-g_{p, p+\nu }(\tau _2) \le 0$ when *p* is odd (see Proposition 4.3).

Since $g_{p, p+\nu }(\tau _2) \le g_{p, p+\nu }(\tau _1)$ (Proposition 4.4), it follows from (35) that $g_{p, p+\nu }(\tau _1') \le g_{p, p+\nu }(\tau _1)$. Using also (34) and the fact that $g_{p, p+\nu }(\tau _1) \ge 0$, we obtain $\Vert x_1' \Vert ^{\nu } g_{p, p+\nu }(\tau _1') \le \Vert x_1' \Vert ^{\nu } g_{p, p+\nu }(\tau _1) \le \Vert x_1 \Vert ^{\nu } g_{p, p+\nu } (\tau _1)$. Thus,

$$\begin{aligned} \Vert x_2 \Vert ^\nu g_{p, p+\nu }(\tau _2) - \Vert x_1 \Vert ^\nu g_{p, p+\nu }(\tau _1) \le \Vert x_2 \Vert ^\nu g_{p, p+\nu }(\tau _2) - \Vert x_1' \Vert ^\nu g_{p, p+\nu }(\tau _1'). \end{aligned}$$

Note that in the right-hand side, we have the difference of the derivatives $D^p f_{p+\nu }(x_2) [h]^p$ and $D^p f_{p+\nu }(x_1')[h]^p$, where the points $x_1'$ and $x_2$ lie on a line, passing through the origin. Therefore, from Theorem 6.2, it follows that

$$\begin{aligned} \Vert x_2 \Vert ^\nu g_{p, p+\nu }(\tau _2) - \Vert x_1 \Vert ^\nu g_{p, p+\nu }(\tau _1) \le C_{p, \nu } \Vert x_2 - x_1' \Vert ^\nu , \end{aligned}$$

which proves (33), in view of (28) and (34).

**Case II(b)** Now suppose $\tau _1 \le \tau _2$. Denote by $\tilde{H}_{p, \nu }$ the constant of $\nu$-Hölder continuity of the polynomial $g_{p, p+\nu }$ on the interval $[-1, 1]$. To prove (33), it suffices to show that

36 $$\begin{aligned}&\Vert x_2 \Vert ^\nu g_{p, p+\nu }(\tau _2) - \Vert x_1 \Vert ^\nu g_{p, p+\nu }(\tau _1) \nonumber \\&\quad \le g_{p, p+\nu }(0) (\Vert x_2 \Vert ^\nu - \Vert x_1 \Vert ^\nu ) + {\tilde{H}_{p, \nu }} ( \Vert x_2 \Vert \tau _2 - \Vert x_1 \Vert \tau _1 )^\nu , \end{aligned}$$

Indeed, recall that $\Vert x_2 \Vert ^\nu - \Vert x_1 \Vert ^\nu \le \Vert x_2 - x_1 \Vert ^\nu$. Also

$$\begin{aligned} ( \Vert x_2 \Vert \tau _2 - \Vert x_1 \Vert \tau _1 )^\nu = \langle B (x_2 - x_1), h \rangle ^\nu \le \Vert x_2 - x_1 \Vert ^\nu \end{aligned}$$

by the Cauchy–Schwartz inequality. Therefore, if (36) is true, then

$$\begin{aligned} \Vert x_2 \Vert ^\nu g_{p, p+\nu }(\tau _2) - \Vert x_1 \Vert ^\nu g_{p, p+\nu }(\tau _1) \le (g_{p, p+\nu }(0) + {\tilde{H}_{p, \nu }}) \Vert x_2 - x_1 \Vert ^\nu , \end{aligned}$$

where $g_{p, p+\nu }(0) + \tilde{H}_{p, \nu } \le A_ {p, \nu }$ in view of Proposition 4.2, Theorems 5.1 and 5.2. Thus, it remains to show (36), or, equivalently, that

$$\begin{aligned}&\Vert x_2 \Vert ^\nu (g_{p, p+\nu }(\tau _2) - g_{p, p+\nu }(0)) \\&\quad \le \Vert x_1 \Vert ^\nu (g_{p, p+\nu }(\tau _1) - g_{p, p+\nu }(0)) + {\tilde{H}_{p, \nu }} ( \Vert x_2 \Vert \tau _2 - \Vert x_1 \Vert \tau _1 )^\nu . \end{aligned}$$

Denote $\rho := \frac{\Vert x_1 \Vert }{\Vert x_2 \Vert } \in [0, 1]$. We need to prove that

37 $$\begin{aligned} g_{p, p+\nu }(\tau _2) - g_{p, p+\nu }(0) \le \rho ^\nu (g_{p, p+\nu }(\tau _1) - g_{p, p+\nu }(0)) + {\tilde{H}_{p, \nu }} ( \tau _2 - \rho \tau _1 )^\nu . \end{aligned}$$

Note that the right-hand side of this inequality, as a function of $\rho \in [0, 1]$, is *concave* (and well-defined, since $\tau _1 \le \tau _2$). Hence, to justify (37), we only need to prove the following two boundary cases:

$$\begin{aligned}&\rho =0: \quad g_{p, p+\nu }(\tau _2) - g_{p, p+\nu }(0) \le {\tilde{H}_{p, \nu }} \tau _2^\nu . \\&\rho =1: \quad g_{p, p+\nu }(\tau _2) - g_{p, p+\nu }(\tau _1) \le {\tilde{H}_{p, \nu }} (\tau _2-\tau _1)^\nu . \end{aligned}$$

But both of them follow from the definition of ${\tilde{H}_{p, \nu }}$.$\square$

Comparing the result of Theorem 6.3 with the lower bound $C_{p, \nu }$, given by Theorem 6.1, we see that for *odd* values of *p*, the constant $\tilde{A}_{p, \nu }$ is *optimal*. Unfortunately, this is no longer true for *even* values of *p*. Nevertheless, the constant $\tilde{A}_{p, \nu }$ is still quite accurate. Indeed, since

$$\begin{aligned} (p-1)!! = \prod _{i=1}^{p/2} (2i-1) \le \prod _{i=1}^{p/2} (\nu +2i-1), \end{aligned}$$

we have

$$\begin{aligned} (p-1)!! \prod _{i=1}^{p/2} (\nu +2i) \le \prod _{i=1}^p (\nu +i). \end{aligned}$$

Thus, the constant $\tilde{A}_{p, \nu }$ is at most two times suboptimal: $\tilde{A}_{p, \nu } \le 2 C_{p, \nu }$.

One may think that the reason, why we obtained a suboptimal bound for even values of *p*, is related to the fact that we had used a suboptimal value for the Hölder constant $H_{p, \nu }$ of the polynomial $g_{p, p+\nu }$ (see the corresponding discussion at the end of Sect. 5). However, this is not the actual reason. Indeed, let us look what happens when we use the optimal value for $H_{p, \nu }$ in the particular case $p=2$. Recall that the optimal constant in this case is

$$\begin{aligned} H_{2, \nu } = \nu (\nu +2) \frac{2^{2-\nu } (1-\nu )^{1-\nu }}{(2-\nu )^{2-\nu }}. \end{aligned}$$

Substituting this expression into (27), we obtain an improved estimate

$$\begin{aligned} A_{2, \nu } = \nu +2 + H_{2, \nu } = (\nu +2) \left( \nu +1 + \nu \frac{2^{2-\nu } (1-\nu )^{1-\nu }}{(2-\nu )^{2-\nu }} \right) . \end{aligned}$$

However, this new estimate is still different from the lower bound

$$\begin{aligned} C_{2, \nu } = (\nu +1) (\nu +2). \end{aligned}$$

At the same time, for small values of $\nu$, the difference between $A_{p, \nu }$ and $C_{p, \nu }$ is almost negligible.

## Lipschitz Constants of Derivatives of Powers of Euclidean Norm

For even values of *p*, our estimate $A_{p, \nu }$ of the Hölder constant of $D^p f_{p+\nu }$ was suboptimal. It turns out that in the special case when $\nu =1$, it is actually very simple to eliminate this drawback and obtain an optimal constant for *all* values of *p*. This case corresponds to *Lipschitz* continuity.

### Theorem 7.1

For any integer $p \ge 0$, the derivative $D^p f_{p+1}$ is Lipschitz continuous with constant

$$\begin{aligned} C_{p, 1} = (p+1)!, \end{aligned}$$

where *n*! for a nonnegative integer *n* denotes the factorial of *n*.

### Proof

It suffices to prove that $| D^{p+1} f_{p+1}(x)[h]^{p+1} | \le (p+1)!$ for all $x \in \mathbb {E}$ and all unit $h \in \mathbb {E}$. By Theorem 3.1, we have $D^{p+1} f_{p+1}(x)[h]^{p+1} = g_{p+1, p+1}(\tau _h(x))$. Since $|\tau _h(x)| \le 1$, we obtain $| D^{p+1} f_{p+1}(x)[h]^{p+1} | \le \max _{[-1, 1]} | g_{p+1, p+1} |$. The claim now follows from Proposition 4.5.$\square$

## Conclusions

In this work, we have proved that derivatives of powers of Euclidean norm are Hölder continuous and have obtained explicit expressions for the corresponding Hölder constants. We have shown that our constants are optimal for odd derivatives and at most two times suboptimal for the even ones. In the particular case of integer powers, when the Hölder condition corresponds to the Lipschitz condition, we have managed to improve our result and obtained optimal constants in all cases. We believe that in general, it should be possible to obtain optimal constants for even derivatives as well. However, this seems to be a difficult problem.

## Footnotes

1 For a positive integer *n*, by *n*!! we denote the double factorial of *n* (the product of all integers between 1 and *n* with the same parity as *n*). We also define $(-1)!! = 0!! = 1$.
